# Dance-based exercise interventions in older adults: a narrative review examining the effects on depression, cognitive-motor function, mood, and quality of life

**DOI:** 10.3389/fpsyt.2026.1862532

**Published:** 2026-08-12

**Authors:** Shanshan Jin

**Affiliations:** Education Science College, Chifeng University, Chifeng, Inner Mongolia, China

**Keywords:** cognitive-motor function, dance-based exercise, depression, mood, older adults

## Abstract

Dance-based exercise has emerged as a promising non-pharmacological approach to support healthy aging by simultaneously engaging cognitive, motor, emotional, and social functions. This narrative review synthesizes evidence from randomized controlled trials and selected clinical studies investigating the effects of various dance modalities, including social dance, Latin dance, square dance, tango, and dance-based exergaming, on depression, cognitive-motor function, mood, and quality of life (QoL) in older adults. Overall, current evidence suggests that dance-based interventions may improve executive function, global cognition, attention, memory, balance, gait, mobility, and lower-limb strength. Several studies also reported improvements in depressive symptoms, mood, social engagement, and QoL. Rhythm- and dual-task-based dance programs have been proposed to enhance cognitive-motor integration and may contribute to improved neuromuscular coordination and sensorimotor performance. However, considerable heterogeneity in intervention characteristics, participant populations, outcome measures, and study quality limits direct comparisons across studies and precludes definitive conclusions regarding the superiority of dance over conventional exercise or the underlying neurobiological mechanisms. Overall, dance-based exercise appears to be a feasible and enjoyable multimodal intervention with the potential to benefit cognitive, motor, and psychosocial health in older adults. Nevertheless, further well-designed, adequately powered, and longitudinal studies with standardized intervention protocols are needed to clarify its long-term effectiveness and the mechanisms underlying its potential benefits.

## 1 Introduction

The phenomenon of population aging is one of the most significant demographic trends of the twenty-first century and is associated with an increasing prevalence of age-related cognitive, motor, and psychological impairments (1). Age-related declines in executive function, memory, attention, and processing speed frequently occur together with impairments in balance, gait, and coordination, thereby increasing the risk of falls, disability, and loss of independence among older adults (2, 3). Emerging evidence suggests that cognitive and motor impairments are closely interconnected through shared neural networks involving frontal, parietal, cerebellar, and subcortical brain regions (4, 5). Furthermore, aging is associated with an elevated incidence of affective disorders, including depression and anxiety, alongside a decline in overall quality of life (QoL) (6, 7). Collectively, these age-related changes impose an increasing burden on healthcare systems and highlight the need for effective, accessible, and sustainable interventions that simultaneously target cognitive, motor, and psychosocial health (8). Pharmacological therapies remain an important component of managing cognitive impairment and mood disorders in older adults (9). However, their effectiveness is often modest, particularly during the early stages of cognitive decline, and long-term treatment may be limited by adverse effects, polypharmacy, and reduced adherence (10). Likewise, traditional exercise regimens, including aerobic and resistance training, have demonstrated benefits for physical and cognitive health. Nevertheless, these interventions primarily target physical performance and may provide less cognitive, emotional, and social stimulation than multimodal activities, potentially limiting long-term engagement and adherence in some older adults (11, 12).

In response to the limitations of pharmacological and conventional exercise approaches, there has been growing interest in multimodal interventions that integrate physical, cognitive, and social components within a single activity. Such interventions are grounded in the concept of neuroplasticity, referring to the brain’s capacity to adapt structurally and functionally in response to complex and enriched experiences (13). Engagement in cognitively demanding and motorically complex activities may support brain health by promoting functional connectivity across distributed neural networks (14). Activities that combine motor coordination, cognitive effort, sensory processing, and social interaction are considered particularly beneficial, as they simultaneously engage multiple brain systems. This aligns with the cognitive-motor dual-task framework, which suggests that performing simultaneous cognitive and motor tasks can enhance executive control and attentional resources, particularly in aging populations (15). Sensorimotor integration, defined as the ability to integrate sensory feedback with motor output, is also increasingly recognized as a key mechanism underlying mobility and balance in older adults (16). Moreover, socially and emotionally engaging activities have been associated with improved adherence, mood regulation, and psychological well-being in older adults (17). Within this context, dance-based exercise has emerged as a specific form of multimodal intervention that incorporates rhythmic movement, cognitive sequencing, musical cues, and social interaction within a structured physical activity setting (18, 19). Unlike conventional exercise, dance requires continuous adaptation to rhythm and space, as well as memory, attention, and executive control processes. This combination of cognitive, motor, and emotional demands may make dance particularly suitable for promoting adherence and engagement in older adults. Recent studies suggest that dance-based interventions may contribute to improvements in multiple domains of aging, including balance, gait, functional mobility, and cognitive performance, although the strength of evidence varies across studies (20, 21). In addition, some studies have reported improvements in mood, depressive symptoms, social participation, and QoL following participation in structured dance programs (18).

Despite the growing body of evidence supporting the benefits of dance-based interventions, important gaps remain regarding their overall effectiveness, optimal intervention characteristics, and the mechanisms underlying their beneficial effects in older adults (22–24). Existing studies vary considerably in terms of study design, sample characteristics, intervention protocols, dance styles, duration, intensity, and outcome measures, which limits the ability to draw firm and consistent conclusions across studies (20, 24). In addition, the methodological heterogeneity and variability in reporting quality across trials further complicate direct comparisons and synthesis of findings (25, 26). Although several studies have suggested beneficial effects of dance-based interventions on cognitive, motor, and psychosocial outcomes in older adults, the evidence remains heterogeneous and partially inconsistent, particularly regarding long-term effectiveness and the optimal characteristics of intervention protocols, including dance modality, intensity, frequency, and duration (23, 24). Accordingly, the objective of this narrative review is to systematically and critically synthesize current evidence on dance-based exercise interventions in older adults, with a specific focus on depression, cognitive-motor function, mood, and QoL. This review also aims to highlight methodological limitations in the existing literature and identify directions for future research in this emerging field.

## 2 Methods

### 2.1 Review design

This narrative review was designed to provide a critical and integrative synthesis of the existing literature, with the aim of identifying emerging themes, interpreting diverse findings, and highlighting knowledge gaps. Unlike systematic reviews that typically follow predefined protocols for study identification, selection, and appraisal, this review focused on a broader conceptual interpretation of the evidence.

### 2.2 Search strategy

A literature search was conducted in PubMed, Scopus, and Web of Science to identify relevant studies published in English up to March 2026. The search combined Medical Subject Headings (MeSH), where applicable, and free-text keywords related to dance-based interventions and healthy aging. The primary search terms included: (“dance” OR “dance-based exercise” OR “dance therapy” OR tango OR “social dance” OR “square dance” OR exergaming) AND (“older adults” OR elderly OR aging) AND (cognition OR “cognitive function” OR “executive function” OR depression OR mood OR “quality of life” OR balance OR gait OR Parkinson* OR dementia). Additional relevant publications were identified through manual screening of the reference lists of eligible articles. Priority was given to randomized controlled trials, controlled clinical studies, and high-quality review articles examining cognitive, motor, psychological, and psychosocial outcomes associated with dance-based interventions in older adults. This narrative review was not designed as a systematic review and therefore did not involve a registered protocol, formal risk-of-bias assessment, or quantitative synthesis. However, key principles of transparent reporting, including description of the literature search strategy and study selection rationale, were incorporated to improve methodological clarity.

## 3 Conceptual framework of dance-based exercise

### 3.1 Definition of dance-based interventions

Dance-based exercise can be defined as a form of physical activity that combines structured or semi-structured movement with rhythm, music, and often social interaction (27, 28). Dance-based interventions can include a variety of modalities such as social and ballroom dance, Argentine tango, contemporary dance, and culturally specific folk dances, as well as emerging digital formats such as dance-based exergaming. Compared to conventional exercise, dance typically involves the simultaneous engagement of motor, cognitive, and perceptual processes, including attention, memory, sequencing, and spatial awareness (29). This integrated cognitive–motor demand may contribute to both physical and cognitive stimulation, although the magnitude of these effects can vary depending on intervention type and intensity. In addition, dance-based interventions can be adapted to different levels of physical ability and implemented in both group and individual settings, which may enhance accessibility and adherence among older adults. Overall, dance represents a multimodal activity that combines physical, cognitive, and social components, and may offer potential benefits for physical and psychosocial health in aging populations, although outcomes remain heterogeneous across studies.

### 3.2 Cognitive-motor dual-task theory

The cognitive–motor dual-task framework provides an important theoretical basis for understanding how dance-based interventions may simultaneously engage motor control, executive function, attention, and cognitive processing in aging and neurodegenerative populations (30–32). This theory suggests that simultaneous engagement in cognitive and motor tasks places demands on attentional control and executive functions, which are known to decline with aging, potentially affecting gait, balance, and functional mobility. Dance-based activities naturally incorporate dual-task conditions, as individuals are required to coordinate complex movements while processing rhythm, adapting to external cues, and recalling movement sequences. Studies comparing externally guided versus internally guided dance movements have shown that different modes of attentional control may differentially influence mobility, cognitive performance, and psychosocial outcomes, particularly in individuals with Parkinson’s disease (PD) and freezing of gait (33). Neuroimaging evidence from multisensory dance training further suggests that such interventions may engage distributed brain networks involved in motor planning, sensory integration, and executive control, supporting the neural basis of dual-task performance (34). In addition, interventions combining music and structured movement have been associated with improvements in executive function and cognitive performance, indicating that rhythm-based training may support cognitive–motor interaction processes relevant to dual-task performance (35). From a broader perspective, neuroplasticity-based frameworks propose that repeated engagement in complex dance activities may support adaptive changes in brain networks involved in cognitive–motor integration across both aging and neurological conditions (36). Beyond cognitive and motor outcomes, group-based dance activities may also enhance social connectedness and sense of community, which can indirectly support motivation, adherence, and sustained engagement in dual-task training contexts (37). Overall, dance-based interventions may represent an ecologically valid form of dual-task training that integrates cognitive, motor, emotional, and social components. However, the available evidence remains heterogeneous, and further high-quality controlled studies are needed to clarify long-term effects and underlying mechanisms.

### 3.3 Rhythm, music, and sensorimotor integration

Rhythm and music play an important role in dance-based exercise by supporting sensorimotor integration and improving movement coordination. Auditory rhythmic stimuli provide a temporal structure that may facilitate movement synchronization through auditory–motor coupling mechanisms involving cortical and subcortical motor networks (38, 39). This synchronization between rhythmic cues, motor execution, and cognitive processing has been associated with improvements in movement timing, gait performance, and coordination, particularly in older adults (23, 40). In addition, music engages emotional and reward-related neural systems, which may contribute to increased motivation and adherence to physical activity (41). Multisensory stimulation through the integration of music, rhythmic cues, and movement may support cognitive processes such as attention, memory, and executive function; however, the available evidence remains heterogeneous due to variations in intervention protocols, participant characteristics, and outcome measures (21, 23, 24). Rhythmic entrainment has been shown to have beneficial effects on motor performance and rehabilitation outcomes, especially in individuals with neurological conditions (38, 42). Overall, the integration of rhythm and music in dance-based interventions may provide a meaningful multimodal stimulus for motor and cognitive engagement in aging populations.

### 3.4 Neuroplasticity mechanisms

Dance-based physical activity has been suggested to promote neuroplastic adaptations by engaging distributed neural networks involved in cognitive and motor functions. During dance practice, brain regions associated with executive control, attentional regulation, motor planning, and visuospatial processing, including the prefrontal cortex, may be recruited to support movement planning, coordination, and adaptive decision-making during complex movement sequences (21, 43, 44). The hippocampus is involved in memory formation and spatial navigation, particularly during learning and recall of movement sequences (21, 45). The basal ganglia play an important role in motor coordination, rhythm processing, and procedural learning, functions that may be repeatedly recruited during rhythmic and sequential movement patterns characteristic of dance-based interventions (21, 43, 46). Overall, these distributed neural processes may support functional brain adaptations associated with complex motor–cognitive training in older adults. Some evidence suggests that long-term engagement in dance or other cognitively enriched physical activities may be associated with structural and functional brain adaptations in older adults, including alterations in functional connectivity and preservation of brain regions involved in cognitive and motor functions (21, 47); however, these findings remain heterogeneous and are primarily derived from small-scale or exploratory studies (21). Compared with conventional exercise, dance may provide a more complex multimodal stimulus due to its integration of cognitive, motor, emotional, and social components, although direct comparative evidence remains limited.

### 3.5 Social and affective engagement pathways

Dance-based physical activity integrates important social and emotional components that may contribute to psychological well-being in older adults. Participation in group and partner dance provides opportunities for social interaction, communication, and shared experience, which may help reduce feelings of loneliness and improve mental health outcomes (17, 48). The expression of emotions through movement and music has also been associated with improvements in mood and reductions in symptoms of depression and anxiety, although the magnitude of these effects may vary across populations and intervention types (27). Some evidence suggests that pleasurable and socially engaging activities may engage reward-related neural systems involved in motivation, affective regulation, and social bonding. These processes may involve neurotransmitter pathways related to dopamine and endogenous opioid signaling, although direct evidence linking dance interventions to specific neurotransmitter changes in older adults remains limited (41, 43, 49). In addition, social engagement has been associated with better cognitive functioning and a lower risk of cognitive decline in older adults, supporting the broader role of psychosocial factors in healthy aging (50). Importantly, the enjoyable and interactive nature of dance may enhance motivation, adherence, and long-term participation, which are key factors for sustained benefits in physical activity interventions. Overall, social and emotional pathways likely play an important role in the multidimensional benefits of dance-based exercise, although further high-quality longitudinal studies are needed to clarify these relationships.

## 4 Effects of dance-based exercise and cognitive–motor interventions on physical, cognitive, and functional outcomes in older adults

### 4.1 Evidence from randomized and controlled studies

**Figure 1:**
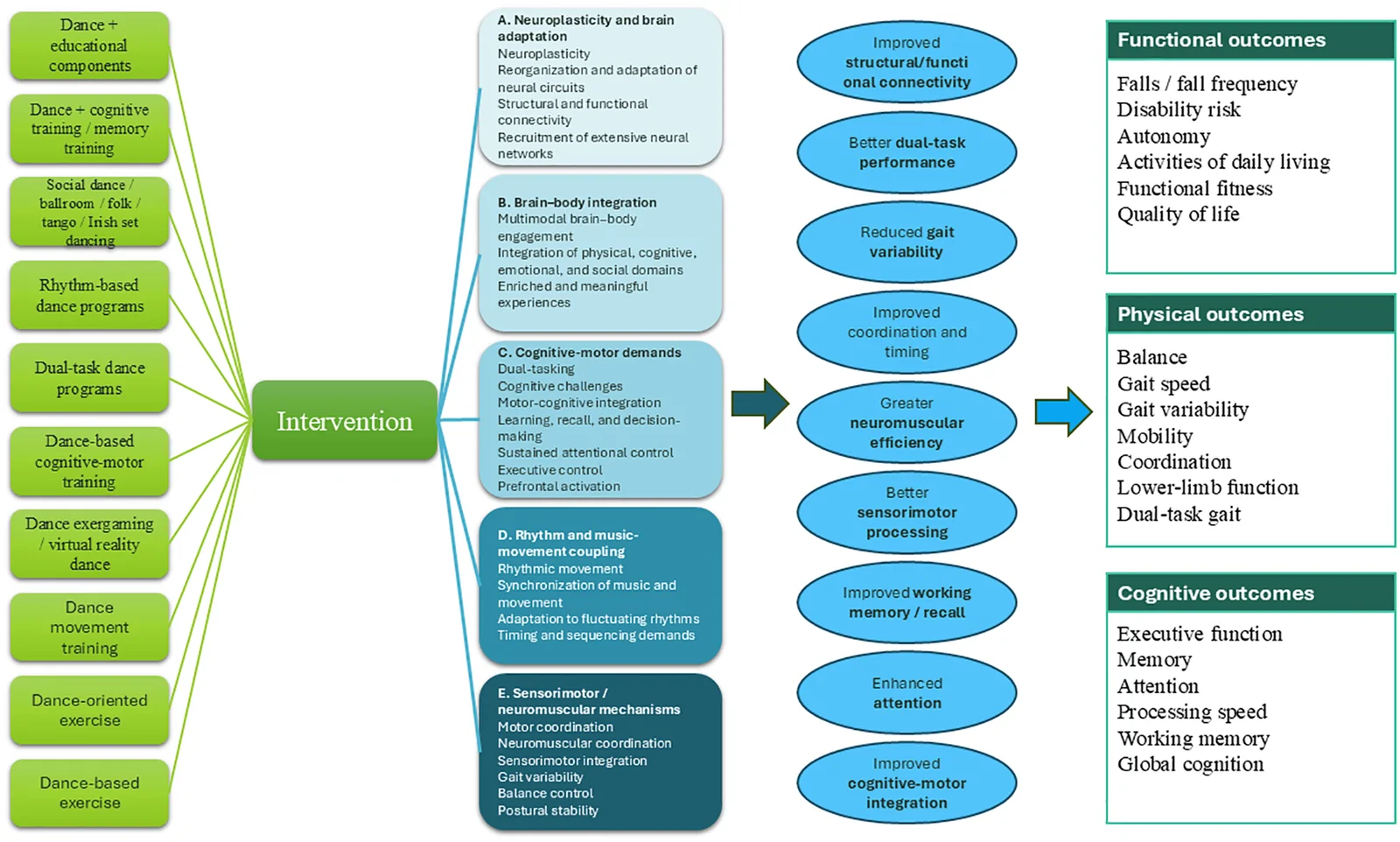
Mechanistic framework of dance-based and cognitive–motor interventions and their effects on cognitive, physical, and functional outcomes in older adults.

A total of 15 randomized and controlled studies evaluating the effects of dance-based interventions on cognitive, motor, psychological, and functional outcomes in older adults were identified and are summarized in **Table 1** and **Figure 1**. The included studies comprised randomized controlled trials, cluster-randomized trials, and controlled intervention studies involving a range of dance modalities, including social dance, ballroom dance, square dance, dance exergaming, dance movement training, and culturally adapted dance programs. The main characteristics and findings of each study are presented below.

**Table 1:** Summary of randomized and controlled trials investigating dance-based interventions on physical, cognitive, and functional outcomes in older adults.

Participants	Intervention	Duration	Outcomes measured	Findings	Methodological notes	Ref
44 older adults (69.1 ± 4.6 yrs)	Supervised group-based dance classes combined with progressive strength training vs supervised endurance training combined with progressive strength training	12 weeks (2×/week)	Physical performance (TUG, 6-min walk, single-leg stance, sit-to-stand, stair climbing), muscle strength/power, muscle thickness, cognitive function (MMSE, dual-task TUG), QoL, depressive symptoms	Both interventions improved physical and cognitive outcomes, including mobility, strength, muscle power, MMSE, and dual-task performance. No significant between-group differences were observed.	Randomized controlled trial; active comparator; no evidence of superiority of dance over endurance training; limited generalizability	(51)
78 older adults (60–75 yrs)	Groove music–based sports dance (GODA) vs conventional music–based sports dance (CODA) vs control; rhythmically synchronized group-based dance training	12 weeks (3×/week)	Emotional regulation, executive function, neuromuscular coherence (β/γ-band), prefrontal connectivity, sensorimotor integration	GODA improved emotional regulation, working memory, planning, neuromuscular coherence, and prefrontal connectivity compared with CODA and control, particularly under low-intensity conditions.	Randomized controlled trial; three-arm design; intensity-dependent effects; limited generalizability to clinical populations	(52)
150 older adults (60–80 yrs)	Structured group-based square dance and Latin dance compared with Tai Chi, Baduanjin, and health education control	12 weeks	Cognitive function (MoCA), strength, flexibility, balance, agility, body composition, psychological well-being, social interaction	Square dance and Tai Chi improved cognitive function; square dance improved strength and endurance; Latin dance improved flexibility and body composition; psychological well-being improved across exercise groups.	Randomized controlled trial; multi-arm design; variability across exercise modalities; domain-specific effects	(53)
89 older adults (34 healthy, 55 MCI)	Supervised group-based dance program for individuals with MCI; community-based rhythmic dance intervention compared with passive control	6 months (2×/week)	Minimum toe clearance (MTC) variability, mean MTC, gait stability, fall-related gait characteristics	Older adults with MCI showed greater MTC variability compared with healthy controls. The dance intervention did not significantly improve MTC variability or mean MTC after the intervention period.	Randomized controlled trial; MCI-specific population; objective gait assessment using inertial sensors; null effects on primary gait outcomes; need for individualized intervention approaches	(54)
88 healthy older adults	Dance-based dual-task aerobic training vs Nordic walking aerobic exercise vs control; home-based group exercise approach incorporating cognitive–motor demands	4 weeks (3×/week, 30 min)	Executive function, global cognition, gait speed, imitation ability, neurological function, muscle mass/output	Both exercise interventions improved executive function. Dance training additionally improved global cognition, gait speed, imitation ability, and selected neurological functions compared with control and showed greater cognitive benefits than Nordic walking. No significant effects were observed on muscle mass or output.	Single-blind randomized controlled trial; short-term intervention; healthy older adults without dementia or sarcopenia; limited follow-up; cognitive–motor design may contribute to observed effects	(55)
57 older Latino adults	Culturally adapted BAILAMOS™ dance program consisting of structured Latin dance sessions compared with health education control	Pilot study	Physical activity levels, cardiorespiratory health, global cognition	The BAILAMOS™ program increased leisure-time moderate-to-vigorous physical activity and improved global cognition compared with health education. Higher attendance was associated with greater improvements in physical activity outcomes.	Pilot randomized controlled trial; culturally adapted dance intervention; adherence-dependent effects; limited generalizability beyond the studied population	(56)
62 older adults (mean 67.5 yrs)	Dance/Movement Training (DMT) compared with aerobic exercise training (AET) and control; structured dance-based movement sessions	12 weeks (3×/week)	Cognition, physical fitness, cardiovascular fitness, mobility, health-related QoL	DMT and AET improved cognitive outcomes and mobility. Cardiovascular fitness improvements were mainly observed in AET, with no clear superiority of DMT over AET for most outcomes.	Randomized controlled trial; active comparator design; open-label intervention; moderate methodological quality; no clear between-group superiority	(57)
82 older adults (≥60 yrs)	Senior Dance (DanSE), a supervised group-based dance program combined with fall-prevention education, compared with education alone	12 weeks (2×/week)	Balance, mobility, gait performance, sit-to-stand ability, cognitive function	Senior Dance improved balance, standing stability, sit-to-stand performance, and walking speed compared with education alone. No significant between-group differences were observed in cognitive outcomes.	Randomized controlled trial; active control condition; assessor-related limitations due to lack of participant and therapist blinding; balance-focused intervention	(58)
530 older adults (mean 78 yrs)	Standardized social dancing program including folk and ballroom dancing delivered in community retirement village settings vs usual activities	12 months (2×/week)	Falls incidence, executive function (Trail Making Test), postural sway, proprioception, reaction time, leg strength, physical performance, gait speed, QoL	Social dancing did not reduce fall incidence or improve most fall-related cognitive and physical risk factors compared with usual activities. Exploratory analyses suggested possible gait speed improvements among ballroom dance participants.	Cluster randomized controlled trial; large community-based study; standardized delivery across sites; moderate adherence; subgroup analyses limited by statistical power	(59)
24 older adults (mean age 80.8 yrs)	Low-dose recreational dance intervention compared with an active non-dance control program in a residential care setting	10 weeks (45 min/week) + 5-month follow-up	Short-term memory, executive function, self-efficacy, life satisfaction	Dance training improved short-term memory and executive function compared with active control, with benefits maintained at follow-up.	Randomized controlled trial; very small sample; active control design; exploratory cognitive training approach; limited statistical power	(60)
32 older adults	Motor–cognitive dance training combining rhythmic movement, coordination, and executive-demanding tasks compared with health-related exercise training	Not specified	Local dynamic stability, gait variability, trunk movement variability during walking	Dance training improved local dynamic stability of walking compared with conventional exercise, whereas no significant changes were observed in gait variability.	Randomized controlled design; blinded outcome assessment; very small sample; objective gait analysis; limited reporting of intervention protocol details	(61)
89 older adults (>70 yrs)	Virtual reality video game dancing (DANCE) combined with strength and balance exercises compared with treadmill walking plus verbal memory training (MEMORY) and treadmill walking alone (PHYS)	6 months + 1-year follow-up	Dual-task gait performance, gait variability, step timing, functional fitness (SPPB, 6-min walk), fall frequency	Cognitive–physical interventions (DANCE/MEMORY) improved selected dual-task gait outcomes compared with physical training alone. Dance-specific adaptations included reduced step time during fast walking, while overall training reduced fall frequency and improved functional fitness.	Randomized controlled trial; multi-arm cognitive–motor design; long-term follow-up; combined intervention components limit attribution of effects specifically to dance	(62)
89 older adults (>70 yrs)	Virtual reality video game dancing (DANCE) combined with strength and balance exercises compared with treadmill walking plus verbal memory training (MEMORY) and treadmill walking alone (PHYS)	6 months + 1-year follow-up	Executive function (shifting attention, working memory), episodic memory, processing speed, global cognitive performance	Simultaneous cognitive–physical interventions improved selected executive functions compared with physical training alone. Dance-based training showed specific benefits for working memory compared with MEMORY, while cognitive improvements were partially maintained at follow-up.	Randomized controlled trial; multi-arm design; cognitive–motor intervention; long-term follow-up; limited ability to isolate the independent contribution of dance	(63)
31 older adults (mean 86.2 yrs)	Cognitive–motor training consisting of progressive strength and balance exercises supplemented with dance video gaming compared with strength and balance training alone	12 weeks (2×/week)	Foot placement accuracy, gait performance under single and dual-task conditions, falls efficacy	Dance video gaming combined with cognitive–motor training improved dual-task gait velocity and single-support time compared with conventional strength and balance training. No significant differences were observed in foot placement accuracy or falls efficacy.	Randomized controlled trial; very old adult population; technology-assisted dance intervention; small sample; limited statistical power for secondary outcomes	(64)
Older adults (pilot study)	Cognitive–motor exercise program combining progressive strength and balance training with additional dance video gaming compared with no specific intervention	12 weeks (2×/week)	Voluntary step execution under single- and dual-task conditions, stepping initiation time	Dance video gaming–based cognitive–motor training improved forward and backward step initiation under dual-task conditions compared with control.	Randomized controlled pilot trial; technology-assisted intervention; small sample; limited outcome scope; exploratory evidence requiring confirmation in larger trials	(65)

TUG, Timed Up and Go; MMSE, Mini-Mental State Examination; QoL, Quality of Life; MoCA, Montreal Cognitive Assessment; MCI, Mild Cognitive Impairment; MTC, Minimum Toe Clearance; GODA, Groove music–based sports dance; CODA, Conventional music–based sports dance; DMT, Dance/Movement Training; AET, Aerobic Exercise Training; PA, Physical Activity; LMVPA, Leisure Moderate-to-Vigorous Physical Activity; VR, Virtual Reality; DT, Dual Task; PHYS, Physical Training.

Merom et al. (51) conducted a randomized controlled trial examining the effects of strength training combined with either endurance exercise or dance-based classes on physical and cognitive outcomes in older adults (51). A total of forty-four participants (mean age 69.1 years) were randomly allocated to either a strength training plus dance group or a strength training plus endurance training group. The intervention lasted 12 weeks with two supervised sessions per week. Participants in the dance condition engaged in structured dance-based exercise sessions delivered in a supervised group format, while the comparison group performed endurance-based aerobic training combined with strength training. Both interventions were designed to improve physical fitness and functional capacity in older adults. Outcomes included physical performance measures such as timed up and go (TUG), 6-minute walk test, single-leg stance, 30-second sit-to-stand, stair climbing, one-repetition maximum, muscle power, and vastus lateralis muscle thickness. Cognitive and psychological outcomes, including dual-task TUG, Mini-Mental State Examination (MMSE), QoL, and depressive symptoms, were also assessed.

A randomized controlled trial investigated non-pharmacological interventions targeting cognitive and emotional functioning in older adults. Seventy-eight participants aged 60–75 years were randomly allocated to three groups: groove music combined with sports dance (GODA: Groove-Oriented Dance Activity), conventional music combined with sports dance (CODA: Conventional Dance Activity), or a control group. The intervention lasted 12 weeks with three sessions per week. All interventions were matched for low intensity to ensure comparable physical exertion across groups. Outcomes included emotional regulation, executive functioning, neuromuscular coherence, and functional connectivity in prefrontal brain regions. The findings indicated that the GODA condition was associated with greater improvements in emotional regulation and executive functioning (e.g., working memory and planning) compared with CODA and control conditions. Furthermore, GODA showed enhanced neuromuscular coherence and increased connectivity between prefrontal regions, suggesting improved sensorimotor integration (52). These effects were reported under specific task conditions and should be interpreted in the context of intervention intensity and study design constraints.

A randomized trial examined the effects of Tai Chi, Baduanjin, square dance, and Latin dance on cognitive, physical, and psychosocial outcomes in older adults. A total of 150 participants were allocated to five groups, including a health education control group, over a 12-week intervention. Outcomes included cognitive function, muscular strength, flexibility, balance, agility, body composition, and psychological well-being. The results indicated improvements in cognitive function in the square dance and Tai Chi groups. Square dance was associated with improvements in grip strength and aerobic endurance, while Latin dance was associated with improvements in flexibility and reductions in body fat. Psychological well-being improved across all exercise groups compared with control (53).

A study investigated gait variability and the effects of dance intervention in older adults with mild cognitive impairment (MCI). The sample included 89 participants, comprising 34 healthy controls and 55 individuals with MCI. Participants with MCI demonstrated higher gait variability compared with healthy controls, indicating increased risk of instability during walking. In the randomized controlled trial phase, participants with MCI were assigned to either a six-month dance intervention or a control condition. No statistically significant between-group differences were observed in gait variability outcomes following the intervention period (54).

A single-blind randomized controlled trial investigated aerobic exercise and dance-based dual-task training in older adults during the COVID-19 confinement period. Eighty-eight participants were allocated to Nordic walking, dance-based aerobic training, or a control group over four weeks. Both intervention groups improved executive function compared with control. The dance group additionally showed improvements in overall cognitive performance, gait velocity, and imitation capacity. These outcomes were observed as differential changes across intervention conditions without implying superiority of one modality over another (55).

The BAILAMOS™ randomized controlled trial evaluated a culturally adapted dance intervention in fifty-seven older Latino adults, randomly assigned to either dance intervention or health education control groups. The intervention was associated with increased leisure-time moderate-to-vigorous physical activity and improved cognitive performance compared with control. Higher adherence (≥75%) was associated with greater increases in physical activity levels across participants (56).

A randomized controlled trial compared Dance/Movement Training (DMT), Aerobic Exercise Training (AET), and a control condition in sixty-two sedentary older adults over 12 weeks. Both DMT and AET were associated with improvements in cognitive function. Aerobic fitness improvements were observed only in AET, whereas mobility outcomes (walking speed, TUG) improved across all groups. No clear between-group superiority was observed for mobility outcomes (57).

A randomized controlled trial examined a Senior Dance program in eighty-two community-dwelling older adults, comparing dance plus education with education alone over 12 weeks. The dance group demonstrated significant improvements in balance and mobility outcomes (single-leg stance, standing balance, sit-to-stand performance, walking speed). No significant differences were observed in cognitive outcomes between groups (58).

A large cluster randomized controlled trial evaluated social dance (folk and ballroom) in 530 older adults over 12 months. No statistically significant differences were observed between groups for falls, executive function, or most physical outcomes. Exploratory analyses indicated limited and inconsistent effects on gait velocity and subgroup-specific responses. Adherence was moderate (59).

A randomized controlled trial examined a low-dose dance intervention in very elderly individuals (mean age 80.8 years) residing in assisted living facilities. Twenty-four participants were randomly assigned to dance intervention or active control. The intervention was associated with improvements in short-term memory and executive function compared with control, with effects maintained at follow-up (60).

A randomized controlled trial evaluated a dance-based motor-cognitive intervention in thirty-two healthy older adults. No significant between-group differences were observed in gait variability. However, improvements in local dynamic stability were observed in the dance intervention group compared with the control condition (61).

A randomized controlled trial compared virtual reality dance, treadmill walking with cognitive training, and treadmill walking alone in eighty-nine older adults over six months. Both cognitive-physical intervention groups showed improvements in gait performance, functional fitness, and fall-related outcomes. Improvements in dual-task gait performance were more pronounced in dance and memory training groups. Long-term follow-up showed comparable outcomes across groups (62).

A randomized controlled trial investigated cognitive and physical training using dance-based exergaming in older adults. Participants were assigned to virtual reality dance, treadmill walking with cognitive training, or treadmill walking alone. Both cognitive-physical groups demonstrated improvements in executive function, including attention shifting and working memory, compared with physical training alone. These effects persisted at one-year follow-up (63).

Two randomized pilot trials evaluated dance video gaming combined with cognitive-motor training in older adults. Results indicated improvements in dual-task gait performance, including gait velocity, step initiation, and single-support time under cognitive load conditions. No significant differences were observed in foot placement accuracy or fall efficacy between groups (64, 65).

## 5 Effects of dance-based interventions (including robot-assisted and technology-enhanced approaches) on motor, cognitive, and functional outcomes in PD and dementia

### 5.1 Evidence from randomized and controlled studies

A feasibility pilot investigation assessed a robot-assisted, dance-oriented rehabilitation program in individuals with early-stage PD (66) (**Table 2**). The intervention included sixteen sessions of Irish dance over eight weeks, delivered through an exergaming system incorporating socially interactive robotic support. The robot functioned as a real-time feedback and cueing interface rather than a standalone therapeutic modality, supporting movement accuracy and engagement within a therapist-guided setting. Findings indicated improvements in motor outcomes, including balance, gait, mobility, and overall physical performance as measured by standardized PD scales such as the Performance-Oriented Mobility Assessment, Short Physical Performance Battery (SPPB), and Unified Parkinson’s Disease Rating Scale-III. Improvements in Timed Up and Go (TUG) performance were also reported. Participants further demonstrated high acceptability and usability of the system. These findings suggest that technology-assisted dance approaches may be feasible and acceptable adjunct strategies for supporting motor rehabilitation in individuals with early-stage PD; however, the contribution of the technological component requires further investigation.

**Table 2:** Effects of dance-based interventions, including robot-assisted and technology-enhanced approaches, on motor, cognitive, and functional outcomes in Parkinson’s disease and dementia: Evidence from controlled and feasibility studies.

Participants	Dance modality and intervention characteristics	Duration	Outcomes measured	Findings	Methodological notes	Ref
20 patients with early-stage PD	Robot-assisted Irish dance-based rehabilitation combined with AI-supported exergaming. The intervention incorporated a socially interactive robot providing contextual assistance, movement cueing, and real-time feedback through an avatar-based exercise platform. Sessions were therapist-guided and delivered in small groups (two participants per session).	8 weeks (2×/week)	Motor function (UPDRS-III), balance, gait (TUG), mobility scales, QoL, acceptability	Improvements were observed in balance, gait performance, functional mobility, UPDRS-III scores, and TUG performance. Participants reported positive acceptance and usability of the robotic exergaming system.	Technology-enhanced dance rehabilitation combining Irish dance, exergaming, and robotic assistance appeared feasible and acceptable for improving motor and functional outcomes in individuals with PD.	(66)
31 older adults with dementia (65–93 yrs)	Tango-based dance therapy delivered as a structured social dance intervention and compared with conventional physical exercise. The intervention emphasized rhythmic partner-based movement, balance challenges, mobility training, and social interaction.	3 months	Gait speed, mobility (TUG), ADL, QoL	Tango therapy improved gait speed and attenuated decline in functional mobility compared with physical exercise. Both groups showed decline in activities of daily living, although deterioration was significant mainly in the control group.	Tango-based dance interventions may support mobility-related outcomes and reduce functional decline in older adults with dementia.	(67)
24 PD patients	Irish set dancing compared with routine physiotherapy exercises. The intervention consisted of instructor-led Irish set dancing classes combined with a home exercise program, emphasizing rhythmic stepping, coordination, balance, and motor practice.	6 months	Motor disability (UPDRS), balance, TUG, freezing of gait, QoL	Both interventions were feasible and safe. The dance group demonstrated greater improvements in freezing of gait, balance, and motor disability compared with physiotherapy. Adherence was high and adverse events were comparable between groups.	Irish set dancing represents a feasible and engaging rehabilitation approach for PD, with potential benefits for mobility, balance, and motor symptoms.	(68)

PD, Parkinson’s disease; UPDRS-III, Unified Parkinson’s Disease Rating Scale Part III (motor examination); TUG, Timed Up and Go; POMA, Performance-Oriented Mobility Assessment; SPPB, Short Physical Performance Battery; ADL, Activities of Daily Living; QoL, Quality of Life; IG, Intervention Group; CG, Control Group.

A randomized controlled trial in individuals with dementia compared tango-based dance therapy with conventional exercise programs (67). The dance intervention group demonstrated improvements in gait velocity and functional mobility, whereas the control group showed decline in certain activities of daily living (ADL). However, no strong conclusions regarding superiority can be drawn due to limitations related to sample size and study duration. These findings suggest that structured dance interventions may provide functional benefits in individuals with dementia, although larger trials are required to determine their clinical effectiveness.

Another randomized controlled feasibility trial evaluated Irish set dancing compared with conventional physiotherapy in individuals with idiopathic PD (n=24, 6-month duration) (68). The study aimed to assess feasibility, safety, and adherence, with motor disability (Unified Parkinson’s Disease Rating Scale; UPDRS) as the primary outcome and mobility, balance, freezing of gait, and QoL as secondary outcomes. Both interventions were feasible, safe, and demonstrated high adherence with low dropout rates. The dance group showed greater improvements in freezing of gait, balance, and motor disability compared with physiotherapy. QoL and functional mobility were also assessed using standardized instruments. These findings indicate that dance-based interventions may represent a promising complementary approach for improving selected motor outcomes in individuals with PD, although evidence remains limited by small sample sizes and feasibility-oriented designs.

Collectively, the available randomized and controlled studies indicate that dance-based interventions may contribute to improvements in motor function, mobility, and functional outcomes in neurodegenerative populations. Nevertheless, the evidence remains preliminary due to heterogeneity in intervention protocols, limited sample sizes, and the predominance of feasibility studies.

**Figure 2:**
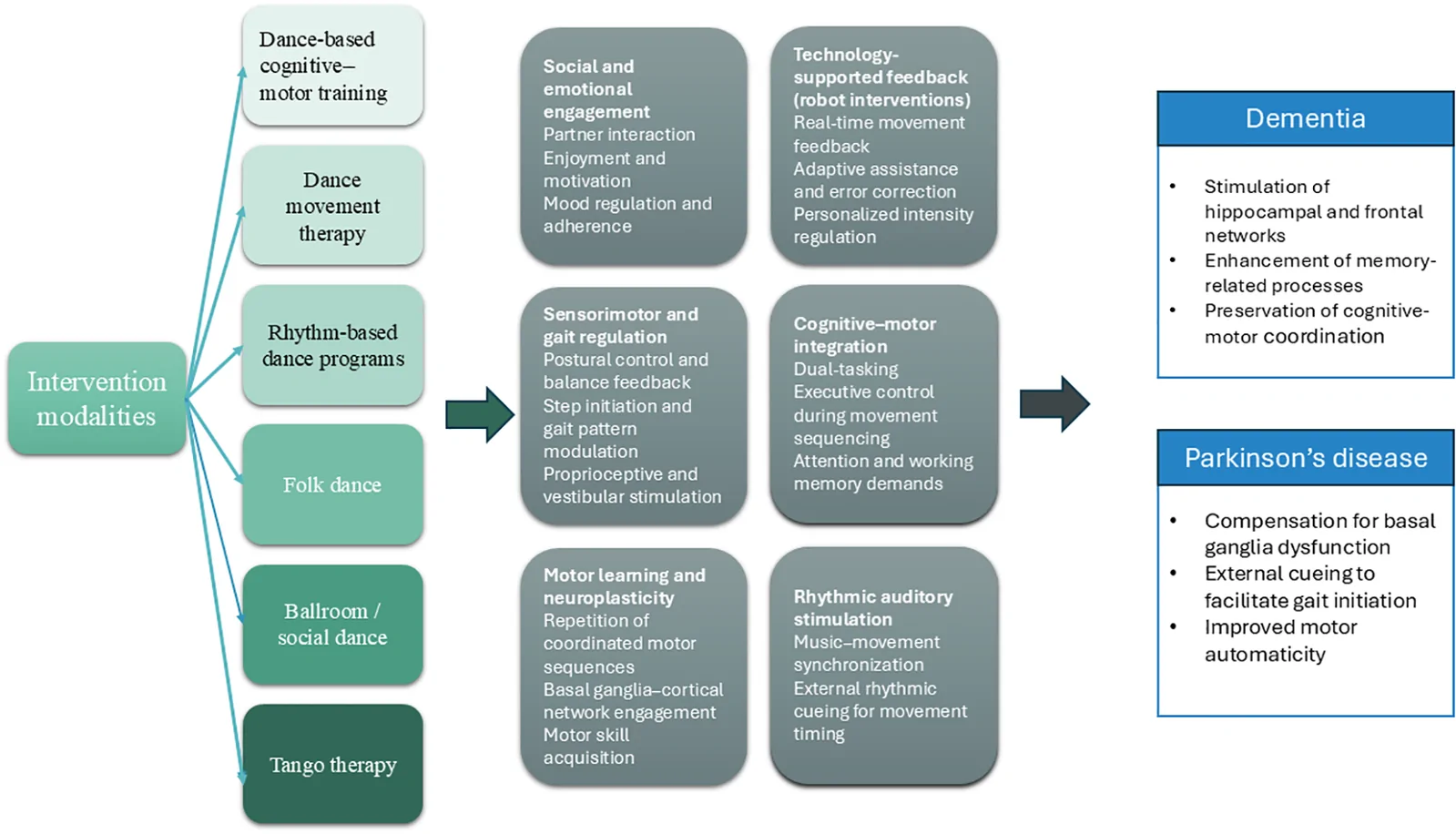
Conceptual model of mechanisms and outcomes of dance-based and robot-assisted dance interventions in Parkinson’s disease and dementia.

Dance-based and cognitive–motor interventions have been proposed to involve multiple interacting processes, including motor–cognitive coupling, sensorimotor integration, and activity-dependent neuroplasticity. Evidence from randomized and controlled studies suggests that these interventions are associated with improvements in executive function, gait performance, balance, and functional mobility in older adults and individuals with neurodegenerative conditions. **Figure 2** provides a conceptual synthesis of the potential mechanisms and outcomes derived from the empirical findings discussed in Section 5.1. Rhythmic auditory–motor synchronization, cognitive demands during movement sequencing, and repeated motor practice may contribute to enhanced sensorimotor coordination and functional performance. However, these proposed mechanisms remain hypothesis-driven, as direct mechanistic evidence from large-scale clinical studies is currently limited.

## 6 Effects of dance-based and exercise interventions on psychophysiological outcomes in older adults

### 6.1 Evidence from randomized and controlled studies

The literature search identified five randomized and controlled studies investigating the effects of dance-based interventions on psychological, cognitive, physical, and functional outcomes in older adults, as summarized in **Table 3**. These studies examined different dance modalities, including folkloric dance, structured rhythmic dance, community-based dance programs, dance movement therapy, and aerobic dance approaches. The findings of each study are described separately below.

**Table 3:** Effects of dance-based and exercise interventions on psychophysiological outcomes in older adults.

Participants	Dance modality and intervention characteristics	Duration	Outcomes measured	Findings	Methodological notes	Ref
40 older women (>65 yrs)	Group-based Turkish folkloric dance program involving structured rhythmic movements, coordinated whole-body activities, and culturally specific dance sequences. The intervention was supervised and designed as a physical activity program targeting balance, mobility, and social engagement.	8 weeks	Physical performance (20-m walk, 6-min walk, stair climbing, chair rise), balance (Berg Balance Scale), depression (GDS), QoL (SF-36)	The dance group showed significant improvements in physical performance, balance, and selected QoL domains compared with baseline and control conditions. No meaningful changes occurred in the control group.	Randomized controlled trial; small sample size; healthy female population only; short intervention duration; limited generalizability to other older populations.	(69)
23 older adults (mean 78 yrs)	Structured dance intervention compared with intensity-matched aerobic exercise. The dance program incorporated rhythmic movement sequences requiring coordination, memory engagement, and social interaction. Both interventions were matched for moderate exercise intensity.	16 weeks (1×/week)	Cognition (MMSE), memory performance, neuropsychiatric symptoms (GDS-15, NPI-R), mobility (TUG), ADL	Dance participants demonstrated improvements in global cognition and verbal memory, whereas aerobic exercise did not produce similar cognitive improvements. Both groups showed reductions in depressive and neuropsychiatric symptoms, with no significant effects on TUG or ADL.	Randomized controlled trial; very small sample size; low-frequency intervention; cognitive impairment population; further studies needed for confirmation.	(70)
Community-dwelling older adults	Community-based structured dance program consisting of repeated group dance sessions combining aerobic activity, rhythmic movement, and coordination-based exercises. The intervention was designed as an accessible lifestyle activity for frailty prevention.	16 weeks (5×/week)	Frailty status, depressive symptoms, SPPB, grip strength	The dance group showed reductions in frailty and depressive symptoms and improvements in physical performance compared with usual activity control.	Randomized controlled trial; high training frequency may limit feasibility in some populations; limited information on long-term adherence and sustainability.	(71)
204 older adults with mild dementia	Dance movement therapy (DMT) involving therapist-guided expressive movement, music-based activities, improvisation, and social interaction. The intervention was compared with intensity-matched conventional physical exercise and waitlist control.	12 weeks + 1-year follow-up	Depression, loneliness, negative mood, daily functioning, neurocognitive outcomes, salivary cortisol measures	DMT improved depressive symptoms, loneliness, negative mood, daily functioning, and cortisol rhythm regulation. Several functional and physiological benefits were maintained at long-term follow-up. The matched exercise group showed limited effects.	Large randomized controlled trial; inclusion of dementia population; long-term follow-up available; mechanisms remain partly exploratory.	(72)
34 older adults (exercise groups) + 11 controls	Low-impact moderate-intensity aerobic dance program with or without additional wrist weights. The intervention combined rhythmic aerobic dance movements with exercises targeting muscular fitness, flexibility, and balance.	10 weeks (3×/week)	Aerobic fitness, muscle strength, flexibility, balance, body composition, mood states	Dance training improved peak oxygen uptake, lower-limb strength, and psychological vigor. Additional wrist weights did not provide extra benefits compared with dance training alone.	Randomized controlled study; moderate sample size; short intervention period; no evidence for long-term outcomes.	(73)

GDS, Geriatric Depression Scale; SF-36, Short Form-36 Health Survey; MMSE, Mini-Mental State Examination; NPI-R, Neuropsychiatric Inventory-Revised; TUG, Timed Up and Go; ADL, Activities of Daily Living; SPPB, Short Physical Performance Battery; QoL, Quality of Life; DMT, Dance Movement Therapy.

A randomized controlled trial evaluated an 8-week Turkish folkloric group dance program in healthy older women aged ≥65 years (69). The intervention consisted of supervised rhythm-based group dance sessions emphasizing coordinated movements and social participation. Compared with the non-exercise control group, participants receiving the dance intervention demonstrated improvements in physical performance, balance-related outcomes, depressive symptoms, and QoL measures. These findings suggest that structured social dance may represent an effective approach for simultaneously targeting physical and psychosocial aspects of healthy aging.

Another randomized controlled study compared dance-based exercise with intensity-matched aerobic exercise in older adults with cognitive impairment over a 16-week intervention period (70). The dance intervention incorporated rhythmic movement sequences requiring coordination, memory, and cognitive engagement. Although both groups showed improvements in depressive and neuropsychiatric symptoms, the dance group demonstrated additional benefits in cognitive outcomes, including global cognition and verbal memory. However, no significant improvements were observed in mobility-related outcomes or activities of daily living, highlighting the domain-specific effects of dance-based interventions.

A 16-week randomized controlled trial investigated a high-frequency community-based dance program in older Chinese adults (71). The intervention involved repeated group dance sessions combining aerobic activity with coordinated movement patterns. Compared with usual activities, participation in the dance program was associated with reductions in frailty and depressive symptoms, together with improvements in physical performance. These findings support the potential role of accessible community-based dance programs in promoting both physical resilience and emotional well-being among older adults.

A randomized controlled trial examined dance movement therapy (DMT) compared with conventional exercise and a waitlist control condition in older adults with mild dementia (72). The DMT intervention emphasized therapist-guided expressive movement, music engagement, improvisation, and social interaction. Compared with control conditions, DMT was associated with improvements in depressive symptoms, loneliness, negative mood, and functional outcomes, with some benefits maintained during long-term follow-up. These findings suggest that the psychosocial and emotionally engaging components of dance may provide additional benefits beyond conventional physical exercise, particularly in populations experiencing cognitive decline.

A controlled study evaluated a moderate-intensity aerobic dance program, with or without additional wrist weights, in older adults (73). The intervention involved structured rhythmic aerobic dance sessions performed regularly over the intervention period. Both dance groups demonstrated improvements in aerobic capacity, lower-limb strength, and mood-related outcomes, whereas the addition of wrist weights did not provide further measurable benefits. These results indicate that the primary benefits of aerobic dance may be achieved through the integrated effects of rhythmic movement, cardiovascular stimulation, and engagement rather than additional external resistance.

Overall, the available evidence indicates that dance-based interventions may positively influence mood, depressive symptoms, cognitive performance, physical function, and QoL in older adults. However, differences in dance modality, intervention intensity, participant characteristics, and outcome measures contribute to heterogeneity across studies and should be considered when interpreting the findings.

## 7 Qualitative synthesis and critical appraisal of dance-based intervention studies

The qualitative synthesis of the included studies identified three major evidence domains: physical and cognitive outcomes in older adults, clinical applications of dance-based interventions in PD and dementia populations, and psychophysiological adaptations associated with dance participation. Although the overall evidence indicates potential benefits of dance-based interventions across multiple domains, the strength of conclusions differs according to population characteristics, intervention design, and methodological rigor.

### 7.1 Evidence from physical and cognitive studies in older adults

Studies investigating healthy older adults or community-dwelling elderly populations generally reported improvements in physical performance, balance, mobility, cognitive function, and psychosocial outcomes following dance-based interventions. However, the interpretation of these findings is limited by substantial variability in intervention characteristics, including differences in dance styles (e.g., folk dance, aerobic dance, therapeutic dance, and structured movement programs), session frequency, intensity, intervention duration, and delivery format. Such heterogeneity makes direct comparison between studies challenging and suggests that the effectiveness of dance may depend on the specific characteristics of the implemented program. Furthermore, although several trials employed randomized controlled designs, many included relatively small sample sizes and short intervention periods, limiting statistical power and the ability to determine long-term sustainability of observed benefits.

### 7.2 Evidence from PD and dementia populations

In individuals with PD and dementia, available studies suggest that dance-based interventions may contribute to improvements in motor function, gait performance, balance, functional mobility, and selected psychosocial outcomes. However, most studies in these clinical populations remain preliminary, with small-scale randomized trials or feasibility designs predominating. Intervention approaches vary considerably, ranging from traditional dance modalities to structured therapeutic programs and technology-supported formats such as exergaming-based interventions. Although technology-enhanced approaches, including robotic assistance and virtual reality components, have demonstrated feasibility in selected studies, their effects should be interpreted within the context of individual intervention designs rather than generalized across all dance-based therapies. Additional limitations include variation in disease severity, outcome measures, intervention intensity, and follow-up duration, which restrict the comparability and clinical translation of findings.

### 7.3 Psychophysiological considerations of dance-based interventions

Beyond physical and clinical outcomes, several studies have explored psychophysiological responses associated with dance participation, including mood regulation, emotional well-being, social engagement, stress-related responses, and neurocognitive adaptations. These findings support the concept that dance represents a multidimensional intervention integrating motor, cognitive, emotional, and social components. Nevertheless, interpretation of psychophysiological outcomes requires caution because many studies rely on self-reported measures of psychological status, which may introduce reporting bias. In addition, objective biomarkers and neurophysiological assessments remain limited and are not consistently incorporated across studies.

### 7.4 Common methodological limitations and future research directions

Across all evidence domains, several common methodological limitations should be considered. Blinding of participants and intervention providers is generally not feasible in exercise- and behavior-based interventions, potentially introducing performance bias. Although randomization was frequently applied, reporting of allocation concealment, adherence monitoring, and protocol fidelity was often insufficient. Attrition may also influence longer-duration studies, particularly those extending beyond six months. Due to the narrative nature of this review, formal risk-of-bias scoring or quantitative quality assessment was not performed; instead, a qualitative synthesis approach was used to contextualize methodological strengths and limitations. Overall, while current evidence supports the potential value of dance-based interventions for improving motor, cognitive, and psychosocial outcomes, larger multicenter randomized controlled trials using standardized protocols, objective outcome measures, and longer follow-up periods are required to establish definitive clinical efficacy.

## 8 Special populations and clinical applications

The studies summarized in **Tables 1**–**3** indicate that dance-based interventions have been investigated across diverse older populations, including individuals with MCI, dementia, PD, frailty, and older adults at risk of functional decline. Overall, the available evidence suggests that dance represents a multidimensional intervention capable of simultaneously engaging motor, cognitive, emotional, and social domains; however, the magnitude and consistency of effects vary according to population characteristics, intervention design, and methodological quality.

In older adults with cognitive impairment or at increased risk of cognitive decline, dance-based interventions have demonstrated potential benefits for cognitive performance, executive function, memory, and mobility-related outcomes (**Table 1**). For example, cognitive–motor dance programs incorporating rhythmic movement and dual-task challenges improved executive function, gait-related performance, and selected cognitive outcomes compared with conventional exercise approaches (55, 62, 63). Similarly, culturally adapted dance interventions such as BAILAMOS™ increased leisure-time moderate-to-vigorous physical activity and were associated with improvements in global cognition, although these findings require confirmation in larger and more diverse populations (56). However, studies targeting gait-related markers in individuals with MCI have produced inconsistent results, indicating that intervention duration, intensity, and individual characteristics may influence responsiveness to dance training (54).

Among individuals with neurodegenerative disorders, particularly PD and dementia, dance-based interventions have shown promising effects on motor function, balance, mobility, and selected aspects of QoL (**Table 2**). In PD populations, Irish set dancing and rhythm-based rehabilitation approaches improved motor disability, freezing of gait, balance, and functional mobility, while demonstrating good adherence and feasibility (68). In addition, technology-supported approaches integrating dance with exergaming and robotic assistance have been explored as innovative rehabilitation strategies, with preliminary evidence indicating improvements in gait, balance, and motor outcomes; however, these findings remain limited to feasibility-level investigations and should not be generalized beyond the specific intervention contexts (66). In older adults with dementia, tango-based dance therapy was associated with improved gait speed and reduced functional decline compared with conventional exercise, suggesting potential value as a socially engaging rehabilitation approach (67).

Psychophysiological studies summarized in **Table 3** further highlight the broader effects of dance participation beyond physical function. Randomized trials have reported improvements in depressive symptoms, emotional well-being, social engagement, and daily functioning following dance-based interventions. For example, dance movement therapy in older adults with mild dementia improved depression, loneliness, daily functioning, and cortisol rhythm regulation, with some effects maintained during long-term follow-up (72). Similarly, structured dance programs in healthy older adults improved balance, physical performance, mood-related outcomes, and selected quality-of-life domains (69–71, 73). These findings support the concept that dance acts as a complex behavioral intervention integrating physical activity, cognitive stimulation, emotional regulation, and social interaction.

Despite these encouraging findings, several limitations should be considered across clinical and at-risk populations. Most available studies remain limited by relatively small sample sizes, short intervention periods, heterogeneity in dance modalities, and variability in outcome assessment (**Tables 1**–**3**). Furthermore, although adherence is frequently reported as favorable, the optimal dose, intensity, and progression of dance interventions remain unclear. Future multicenter randomized controlled trials using standardized intervention protocols, clinically relevant outcomes, and longer follow-up periods are required to establish the clinical effectiveness and mechanisms underlying dance-based interventions across aging-related conditions.

## 9 Limitations of current evidence

Despite the growing interest in dance-based exercise for older adults, several limitations constrain the interpretation of the available evidence. First, substantial methodological heterogeneity exists across studies with respect to dance modality, intervention duration, training frequency and intensity, participant characteristics, comparator interventions, and outcome measures, limiting direct comparisons and reducing confidence in pooled conclusions. Second, most studies have evaluated relatively short-term interventions, leaving the long-term sustainability of cognitive, motor, psychological, and functional benefits insufficiently understood. Third, although improvements in balance, gait, and functional mobility have been consistently reported, these findings have not uniformly translated into significant reductions in fall incidence, particularly in larger randomized trials. Finally, many available studies are pilot or single-center trials with relatively small sample sizes, which may reduce statistical power and limit the generalizability of the findings. Collectively, these methodological limitations suggest that the current evidence is encouraging but should be interpreted with appropriate caution.

## 10 Future directions

Future research should focus on improving methodological rigor and clinical applicability. Standardized intervention protocols, including consistent reporting of dance modality, training intensity, frequency, session duration, and progression, would facilitate comparisons across studies. Larger multicenter randomized controlled trials with longer follow-up periods are needed to determine the durability of intervention effects and identify optimal training parameters. In addition, studies incorporating neuroimaging, wearable technologies, and validated biomarkers may help clarify the biological mechanisms underlying cognitive, motor, and psychological adaptations. Future investigations should also explore personalized approaches that consider differences in functional status, cognitive capacity, and individual preferences, as well as the potential role of technology-assisted and home-based dance programs in improving accessibility and long-term adherence.

## 11 Conclusion

This narrative review indicates that dance-based exercise represents a promising multimodal intervention for older adults, with potential benefits across cognitive, motor, emotional, and quality-of-life outcomes. The combination of physical movement, rhythmic stimulation, cognitive engagement, and social interaction distinguishes dance from many conventional exercise modalities and may contribute to its broad therapeutic potential. Nevertheless, the available evidence remains heterogeneous in terms of intervention characteristics, study design, and outcome assessment, limiting definitive conclusions regarding comparative effectiveness and long-term benefits. Overall, dance-based exercise appears to be a feasible complementary strategy for promoting healthy aging, although further high-quality research is required to establish standardized protocols and strengthen the evidence base for clinical practice.
